# Separation and antioxidant activities of new acetylated EGCG compounds

**DOI:** 10.1038/s41598-023-48387-9

**Published:** 2023-11-28

**Authors:** Jianyong Zhang, Hongchun Cui, Junfeng Yin, Yuwan Wang, Yun Zhao, Jizhong Yu, Ulrich H. Engelhardt

**Affiliations:** 1 Tea Research Institute, Chinese Academy of Agricultural Sciences, Hangzhou, 310008 China; 2 Tea Research Institute, Hangzhou Academy of Agricultural Sciences, Hangzhou, 310024 China; 3 Institute of Food Chemistry, Technischen Universität Braunschweig, Braunschweig, 38106 Braunschweig, Germany

**Keywords:** Biochemistry, Analytical chemistry, Biochemistry

## Abstract

Acetylation could improve the bioavailability of (-)-Epigallocatechin-3-Gallate (EGCG), but the relationship of substitution degree and antioxidant capacity of acetylated EGCG was unclear. The acetylated EGCG products were separated by preparation high performance liquid chromatography (HPLC). Two mono substituted acetylated EGCG, three substituted acetylated EGCG (T-AcE), eight substituted acetylated EGCG (E-AcE) and (-)-Epigallocatechin gallate (EGCG) were isolated. The 7-acetyl-EGCG (S7-ACEGCG) and 7-acetyl-EGCG (T-AcE) were identified for the first time. The antioxidant capacity, superoxide anion radical scavenging capacities, and hydroxyl radical scavenging capacities of EGCG decreased significantly after acetylation modification. The more EGCG acetylation modification sites, the lower the total antioxidant capacity, superoxide anion radical scavenging capacities, and hydroxyl radical scavenging capacities. The antioxidant capacity, superoxide anion radical scavenging capacities, and hydroxyl radical scavenging capacities of 5-acetyl-EGCG (S5-ACE) were higher than 7-acetyl-EGCG (S7-AcE). Combining all the results in this and previous studies, acetylation modification is not conducive to the performance of EGCG antioxidant capacity.

## Introduction

Epigallocatechin gallate (EGCG) is the key bioactive component of tea catechins. Numerous studies showed the beneficial activities of EGCG, including anti-aging, antioxidant, antibacterial, anti-cancer, and so on^1–4^. However, due to the hydroxyl structure of EGCG, the further applications of natural EGCG are restricted, which is the poor lipid solubility, unstable in vivo, low absorption and bioavailability^5–8^. The molecular modification studies showed that the physicochemical properties and biological activity, e.g. anti-aging, antibacterial, anti-cancer of EGCG, could be changed by acylation, esterification, etherification, glycosilation^9–16^. The acetylation molecular modification can effectively improve the bioavailability and biological activity of EGCG^17–19^.

EGCG has eight active hydroxyl sites, and could be replaced by acetyl group^20^. The acetylated EGCG moleculars modified with different degree of substitution are formed by the O-acetylating substitution reaction. Enzymatic synthesis, chemical synthesis, ultrasound-assisted, microwave-assisted techniques are widely used in the preparation of functional active substances^17,19,21–29^. The catechins could been catalyzed by lipase at 45 ℃ for 48 h, and different degrees of acetylcatechins were obtained after purification with silica gel column^30^.

Due to different substituent sites, the chemical characteristics of acetylated EGCG moleculars are also different, such as quantities, space conformation, the anti-cancer, anti radiation, and immuno-enhancement activities. However, the antioxidative activities of different acetylated EGCG moleculars are not clear yet. In this paper, TLC, HPLC/MS and NMR techniques were used to analysis the relationship of substitution degree and antioxidant capacity of acetylated EGCG.

## Materials and methods

### Chemicals

EGCG (> 98%) was supplied by China wuxi taiyogreenpower Technology Co., Ltd. Acetonitrile (chromatography-grade solvents) and methanol (chromatography-grade solvents) were obtained from Germany MERCK Corporation. Ethyl acetate, acetic anhydride and pyridine, ethanol, acetic acid, methylene chloride, sodium bicarbonate, sodium sulfate, sodium chloride obtained from China Beijing Dingguo changsheng Biotechnology Corporation.

### Preparation separation of EGCG acetylating modification

The EGCG sample (1.0 g) was placed into a reactor tube (500 mL). 100 mL of ethyl acetate solution, 2.0 mL acetic anhydride and 1.0 mL of pyridine were added. The reactor tube was put on the magnetic stirrer (MR3001, Heidoph Corporation, Germany). The rotate speed was 400 r/min. The rotate time was 3 h. The 10 mL distilled water was added to stop the reaction. It was allowed to wash by water saturated sodium bicarbonate and saturated sodium chloride. The organic layer was collected and dried with anhydrous sodium sulfate. The coarse EGCG acetylation modified moleculars samples were obtained. Samples were separated by TLC (Scanner4 System, CAMAG Corporation, Switzerland). 4.2 g target coarse sample was obtained.

Coarse sample was separated on an preparative HPLC system (SD1, Varian Corporation, USA) equipped with a pump, an auto sampler, 2487 Dual UV detector and M701 Fraction Collector. Sample was injected into a Sunfire RP-C18 column (30 × 150 mm, paricle size of 5 μm, Sunfire, Waters Corporation, USA). The column temperature was controlled at 35 ℃. Ultra pure water and acetonitrile were used as eluents. The flow rate was 25 mL/min. The detector was set to 280 nm. The following elution profile was used: 0 ~ 100 min, liner gradient from 95 to 65% (v/v) B. The eluant were collected at each branch pipe 20 mL. Fr. 1–Fr. 4 were obtained.

### Analysis of EGCG acetylating compounds

High Performance Liquid Chromatography (HPLC) analysis method: HPLC system (2695-3100SQDMS, Waters Corporation, USA) equipped with a pump, an auto sampler, 2695 detector. Fr. 1–Fr. 4 solution samples were respectively injected into a Sunfire RP-C18 column (2.1 × 100 mm, paricle size of 3 μm, Sunfire, Waters Corporation, USA). The column temperature was controlled at 35 ℃. Ultra pure water containing 0.1% methanoic acid and acetonitrile containing 0.1% methanoic acid were used as eluents. The flow rate was 0.2 mL/min. The detector was set to 280 nm. The following elution profile was used: 0 ~ 15 min, liner gradient from 15 to 40% (v/v) B; 15 ~ 20 min, liner gradient from 40 to 95% (v/v) B; 21 ~ 25 min, isocratic on 95% (v/v) B.

Electrospray ionization-mass spectrometry (ESI–MS) analysis method: Fr. 1–Fr. 4 solution samples were respectively injected into ESI–MS system (Bruke-esquire, Bruke Corporation, Germany). Nitrogen was used as sheath gas and auxiliary gas. Data were collected over the m/z range of 100 ~ 1750. TrapDrive was set as 78.0. Octopole RF Amplitude was set as 150 VPP. Lens was set as − 60.0 V. Capillary Exit was set as 160.0 V. Dry temperature was set as 350. Nebulizer was set as 37 psi. The flow rate of dry gas was 11.00 L/min. HV capillary was set as 4200 V. HV end Plate offset was set as − 500 V. Fr. 2 solution samples were respectively injected into 1H-NMR system (Bruker AM300, Bruke Corporation, Germany) 13C-NMR system (Bruker AM400, Bruke Corporation, Germany). DMSO-d6 was set as the deuterated reagent.

### Antioxidant activity analysis

The antioxidant activities of Fr. 1–Fr. 4 solution samples at 500 μg/mL were studied with total antioxidant capacity (T-AOC) kit, Scavenging superoxide anion capacity Kit, and Scavenging scavenging hydroxyl free radical capacity kit (Nanjing Jiancheng Biological Engineering Institute, China).

### Statistical analysis

Statistical calculations were performed using the SPSS 13.0 for windows release computer program. Results were expressed as mean ± standard deviation (SD). The paired samples two-tailed T-test was applied for the comparison between two groups. Significance was defined as P < 0.05. The statistical analysis of the antioxidant activity data between the means was carried out by one-way analysis of variance (ANOVA) using SPSS statistics 26.0, followed by Duncan’s multiple range test, to compare the means for significant variation (p < 0.05).

## Results and discussion

### Isolation and analysis of EGCG acetylation

**Figure 1:**
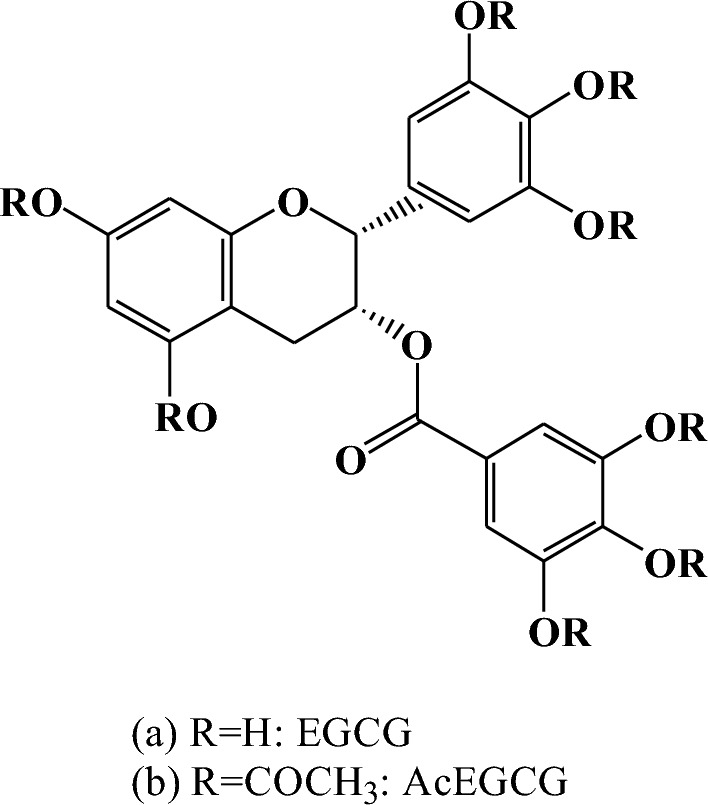
The chemical structure of EGCG (**a**) and AcEGCG (**b**).

The Fr. 1–Fr. 4 was analyzed by HPLC/ESI-MS. The molecular weight of Fr. 1 was 458.4 m/z; MS- base peak data (molecular ion peak [M-H]-) was 457.4 m/z; MS + base peak data (molecular ion peak [M + Na] +) was 481.4 m/z. The above analysis results show that it is EGCG (Fig. 1).

The molecular weight of Fr. 2 was 500.4 m/z. MS + base peak data (molecular ion peak [M + Na] +) was 523.4 m/z. [2 M + Na] + peak data was 1023.8 m/z. MS- base peak data (molecular ion peak [M–H]–) was 499.4 m/z. [2 M–H]– peak data was 999.8 m/z. The molecular weight of EGCG was 458.4 m/z. After acetylation, the hydrogen atoms (molecular weight 1) of a –OH site of the EGCG molecular structure was substituted by a acetyl groups (molecular weight 43). Then the molecular weight of single substituted acetylated EGCG is 500.4 m/z. So, Fr. 2 is a single substituted O-acetylated epigallocatechin gallate (SoEGCG). In the present study, another mono-acetylated EGCG with 501DA has been reported^5^. But the structure elucidation of mono- acetylated EGCG have not revealed. In this study, The infrared spectroscopy data of (a) SoEGCG was following: 3180 (–O–H), 1754 (–C=O), 1673 (–C=C), 1457, 1322 cm^–1^ (Fig. s1a).

**Figure 2:**
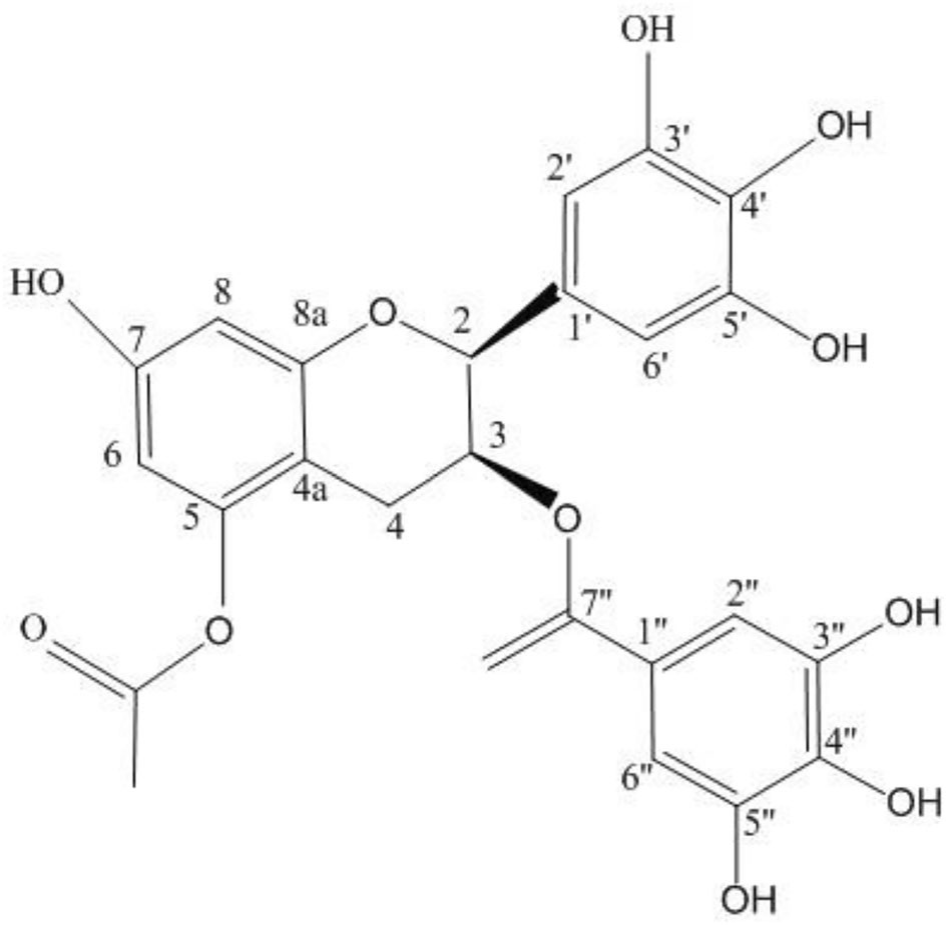
The chemical structure of 5-Acetyl-(-) -EGCG.

In this study, Further ^13^C-NMR and ^1^H-NMR analysis results of Fr. 2 showed that Fr. 2 consists of two substances, including 5-Acetyl-(-)–EGCG (S5-AcE) and 7-Acetyl-(-)–EGCG (S7-AcE). The 13C-NMR result of 5-Acetyl- (-) –EGCG was following: 78.84 (C-2), 69.48 (C-3), 26.83 (C-4), 151.72 (C-5), 102.03 (C-6), 157.26 (C-7), 103.61 (C-8), 130.29 (C-1’), 106.81 (C-2’), 146.72 (C-3’), 133.90 (C-4’), 146.72 (C-5’), 106.81 (C-6’), 121.26 (C-1’’), 110.24 (C-2’’), 146.30 (C-3’’), 139.58 (C-4’’), 146.30 (C-5’’), 110.24 (C-6’’), 167.48 (C=O), 20.96 (AC-Me), 170.78 (AC-C=O). The 1H-NMR result of 5-Acetyl-(-)–EGCG is as follows: δ5.02 (1H, d, J = 1.5 Hz, H-3), δ6.33 (1H, d, J = 1.5 Hz, H-6), δ6.18 (1H, d, J = 1.5 Hz, H-8), δ6.51 (1H, d, J = 1.0 Hz, H-2’), δ6.94 (1H, d, J = 1.5 Hz, H-2’’). Based on the results of 1H-NMR and 13C-NMR spectra, the molecular formula of 5-Acetyl- (-) -EGCG is C_24_H_18_O_12_ (Fig. 2).The structure elucidation of 5-Acetyl- (-) –EGCG has not been reported before.

**Figure 3:**
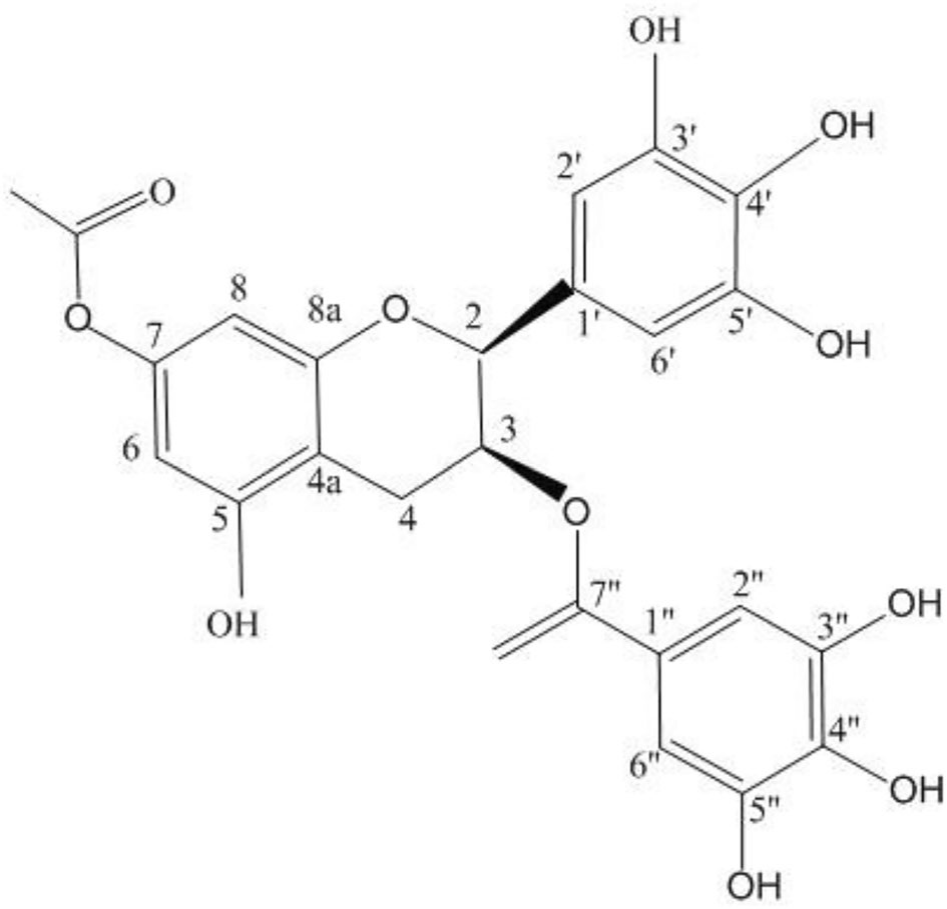
The chemical structure of 7-Acetyl-(-) -EGCG.

The ^13^C-NMR result of 7-Acetyl- (-) –EGCG was following: 8.61 (C-2), 69.17 (C-3), 26.99 (C-4), 157.20 (C-5), 96.51 (C-6), 151.41 (C-7), 95.86 (C-8), 130.29 (C-1’), 106.81 (C-2’), 146.68 (C-3’), 133.90 (C-4’), 146.68 (C-5’), 106.81 (C-6’), 121.26 (C-1’’), 110.24 (C-2’’), 146.30 (C-3’’), 139.58 (C-4’’), 146.30 (C-5’’), 110.24 (C-6’’), 167.48 (C=O), 20.58 (AC-Me), 170.78 (AC-C=O). The 1H-NMR result of 7-Acetyl-(-) -EGCG was follows: δ2.95 (1H, dd, J = 1.5 Hz, H-4), δ6.33 (1H, d, J = 1.5 Hz, H-6), δ6.23 (1H, d, J = 1.5 Hz, H-8), δ6.50 (1H, d, J = 1.0 Hz, H-2’), δ6.92 (1H, d, J = 1.5 Hz, H-2’’). Based on the results of 1H-NMR and 13C-NMR spectra, the molecular formula of 5-Acetyl- (-) -EGCG is C_24_H_18_O_12_ (Fig. 3). The structure elucidation of 7-Acetyl-(-) –EGCG has not been reported before.

The molecular weight of Fr.3 was 484.4 m/z. MS + base peak data (molecular ion peak [M + Na] +) was 507.4 m/z. [2 M + Na] + peak data was 1091.8 m/z. MS- base peak data (molecular ion peak [M–H]–) was 483.4 m/z. [2 M–H]– peak data was 1067.8 m/z. The molecular weight of EGCG was 458.4. After acetylation, the three -OH site (molecular weight 17) of the EGCG molecular structure was substituted by three acetyl groups (molecular weight 129). That is to say, the molecular weight of three acetylated EGCG was exactly 484.4 m/z, which was also the molecular weight of Fr. 3. Then, the Fr. 3 should be three substituted acetylated EGCG. In addition, the 1D/2D NMR data of Fr.3 further confirmed this result. The ^13^C-NMR result of Fr. 3 was following: 77.88 (C-2), 69.97 (C-3), 26.87 (C-4), 157.87 (C-5), 96.77 (C-6), 157.97 (C-7), 95.86 (C-8), 131.45 (C-1’), 124.20 (C-2’), 139.80 (C-3’), 146.35 (C-4’), 115.99 (C-5’), 125.83 (C-6’), 121.44 (C-1’’), 110.18 (C-2’’), 146.35 (C-3’’), 143.34 (C-4’’), 146.35 (C-5’’), 110.18 (C-6’’), 167.59 (C=O), 20.46 (AC-Me). The 1H-NMR result of Fr. 3 was follows: δ5.56 (1H, brs, H-2), δ5.21 (1H, d, J = 1.5 Hz, H-3), δ2.99 (1H, dd, H-4), δ5.98 (1H, s, H-8), δ7.41 (1H, brs, H-2’’), δ6.69 (1H, J = 8.0 Hz, H-5’), δ7.17 (1H, d, J = 8.0 Hz, H-6’), δ6.95(1H, s, H-2’’). Based on the results of MS data and 1H-NMR, 13C-NMR spectra, the Fr.3 is a three substituted O-acetylated epigallocatechin gallate (ToEGCG, T-AcE). In this study, The infrared spectroscopy data of ToEGCG was following: 3371(–O–H), 1709(–C=O), 1617(–C=C), 1400 cm^–1^ (Fig. s1b).

The molecular weight of Fr.4 was 794.4 m/z. MS + base peak data (molecular ion peak [M + Na] +) was 817.4 m/z. [2 M + Na] + peak data was 1611.8 m/z. MS- base peak data (molecular ion peak [M–H]–) was 793.4 m/z. [2 M–H]– peak data was 1587.4 m/z. The molecular weight of EGCG was 458.4. After acetylation, the eight hydrogen atoms (molecular weight 8) of –OH site of the EGCG molecular structure was substituted by three acetyl groups (molecular weight 336 m/z). The molecular weight of eight acetylated EGCG was exactly 794.4 m/z, which was also the molecular weight of Fr. 4. Then, the Fr. 4 should be eight substituted acetylated EGCG. In addition, the 1D/2D NMR data of Fr.3 further confirmed this result. The ^13^C-NMR result of Fr.3 was following: 77.47 (C-2), 71.04 (C-3), 26.30 (C-4), 98.88 (C-5), 96.98 (C-6), 157.93 (C-7), 158.17 (C-8), 135.66 (C-1’), 120.30 (C-2’), 144.70 (C-3’), 138.21 (C-4’), 144.70 (C-5’), 120.30 (C-6’), 129.08 (C-1’’), 123.17 (C-2’’), 144.85 (C-3’’), 140.25 (C-4’’), 144.85 (C-5’’), 123.17 (C-6’’), 164.92 (C=O), 20.40 (2′ 3′ 4′ 5′ -Acetyl), 169.37 (Ace-C=O), 19.98 (3′ 4′ 5′ -Acetyl), 168.13 (Ace-C=O), 20.45(2″ 3″ 4″ 5″ -Acetyl), 169.56 (Ace-C=O). The 1H-NMR result of Fr.3 was follows: δ5.63 (1H, d, J = 1.5 Hz, H-2), δ5.23 (1H, brs, H-3), δ3.07 (1H, dd, H-4), δ5.99 (1H, d, J = 1.5 Hz, H-6), δ6.00 (1H, d, J = 1.5 Hz, H-8), δ7.28 (1H, s, H-2’), δ7.61 (1H, s, H-3’’), δ7.61 (1H, s, H-4’’), δ7.61 (1H, s, H-5’’), δ7.61 (1H, s, H-6’’). In this study, the infrared spectroscopy data of Fr. 4 was following: 3281(–O–H), 1629(–C=O), 1497(–C=C), 1347 cm^–1^ (Fig. s1c). Based on the results of MS data and 1H-NMR, 13C-NMR spectra, IR data, Fr. 4 was a eight substituted peracetylated epigallocatechin gallate (Ep-EGCG, E-AcE) (Fig. 1).

### Total antioxidant capacity of EGCG acetylating components

**Figure 4:**
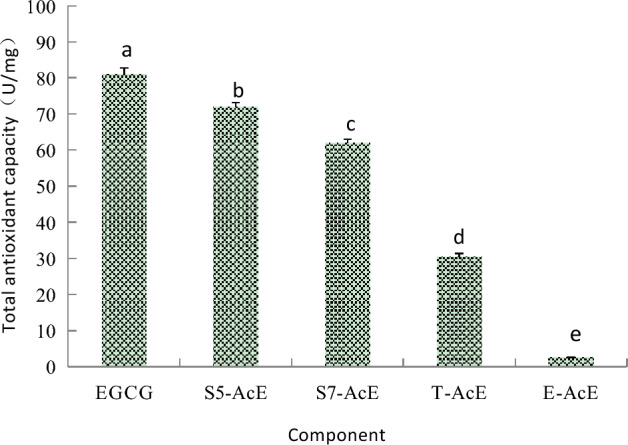
Total antioxidant capacity with different EGCG acetylating separation fraction at 500 μg/mL. Results are expressed as percentage of control and are mean ± SE (n = 6). *P < 0.05, vs. the same concentration of EGCG treated groups.

The total antioxidant capacity kit is mainly to determine the antioxidant ability by restoring Fe^3+^ to Fe^2+^, which combine with phenanthroline for forming a stable complex. The active antioxidant group in molecular structure of EGCG is eight hydroxyl groups, which can not only remove hydroxyl free radicals and reactive oxygen species, decompensate peroxides, block the peroxidation chain, but also can coordinate with metal ions in order to remove metal ion catalysis. The total antioxidant capacities of EGCG, SoEGCG, ToEGCG and AcEGCG are shown in Fig. 4. The total antioxidant capacity of SoEGCG and AcEGCG were lower than EGCG. But The total antioxidant capacity of ToEGCG were higher than EGCG. The ToEGCG in this study, as identified by MS for the first time, had the three -OH site acylated with acetyl groups, and the remaining –OH site on the aromatic rings of EGCG may contribute to the superior antioxidant capacity of ToEGCG. Maintenance of the total antioxidant activity of SoEGCG, ToEGCG and AcEGCG suggest that these derivatives may be used as antioxidants in more application area.

### Scavenging superoxide anion radical capacities of EGCG acetylating components

**Figure 5:**
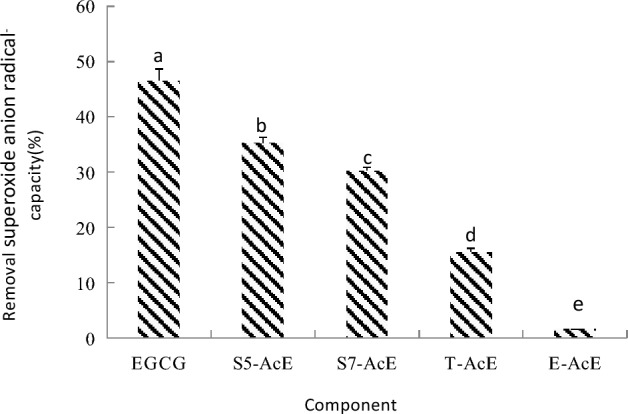
Scavenging superoxide anion capacity with different EGCG acetylating separation fraction at 500 μg/mL. Results are expressed as percentage of control and are mean ± SE (n = 6). *P < 0.05, vs. the same concentration of EGCG treated groups.

The scavenging superoxide anion radical capacities kit is mainly to determine the antioxidant ability by analyzing the reduction on superoxide anion, which generate in the simulated reaction process between medium yellow purine and xanthine oxidase. It is the customary method for antioxidant activity evaluation. The scavenging superoxide anion radical capacities of EGCG, SoEGCG, ToEGCG and AcEGCG are shown in Fig. 5. The scavenging superoxide anion radical capacities of SoEGCG and ToEGCG were lower than EGCG. The scavenging superoxide anion radical capacities of AcEGCG were higher than SoEGCG, ToEGCG, and EGCG. The scavenging superoxide anion radical capacity of SoEGCG was higher than ToEGCG. The eight hydrogen atoms acylated with acetyl groups may contribute to the scavenging superoxide anion radical capacity of AcEGCG.

### Scavenging hydroxyl free radical capacities of EGCG acetylating components

**Figure 6:**
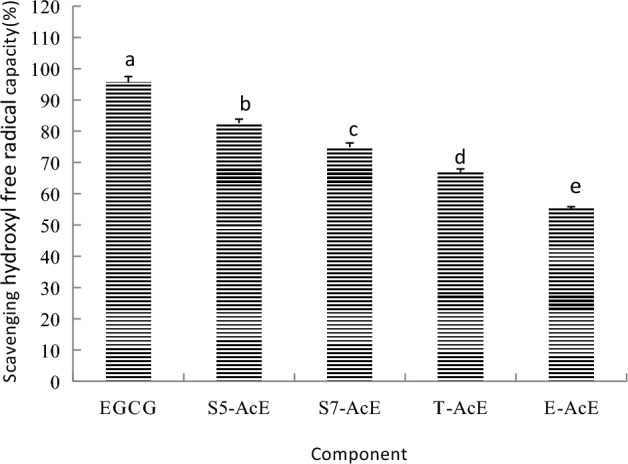
Scavenging hydroxyl free radical antioxidant capacity with different EGCG acetylating separation fraction at 500 μg/mL. Results are expressed as percentage of control and are mean ± SE (n = 6). *P < 0.05, vs. the same concentration of EGCG treated groups.

Scavenging hydroxyl free radical capacity kit, which measures the inhibition degree of absorbance decreasing at 536 nm by samples, is the customary method for antioxidant activity evaluation. The scavenging hydroxyl free radical capacities of EGCG, SoEGCG, ToEGCG and AcEGCG are shown in Fig. 6. The scavenging hydroxyl free radical capacities of SoEGCG, ToEGCG and AcEGCG were lower than EGCG. There may be a certain correlation between acetyl groups space conformation and antioxidant activity. The structure–activity relationship of EGCG acetylating components need further research.

## Conclusions

The preparative liquid chromatography, LC/MS, NMR methods were used to isolate and identify the EGCG acetylating products in this study. The single substituted O-acetylated epigallocatechin gallate (SoEGCG), three substituted O- acetylated EGCG (ToEGCG), and peracetylated EGCG(E-AcEGCG) were isolated. 5-Acetyl-(-)-EGCG and 7-Acetyl-(-)–EGCG were identified. Separation methods in this study, provide the reference for further separation system of EGCG acetylated product, and obtaining different substitution degree of EGCG acetylating components. There are eight hydroxyl groups in the molecular structure of EGCG, which of substitution site contain O-substitution site and C-substitution site. The molecular structure, configuration, conformation and other aspects of different substitution degree of EGCG acetylating components were not clear.

The antioxidant activity of different EGCG acetylating components were different, which were affected not only by the substitution degree, but also by the substitution site. By comparing the total antioxidant activity of peracetylated EGCG and EGCG, it was found that the antioxidant activity of peracetylated EGCG was significantly lower than that of EGCG^17^. The peroxide value (POV) and P- anisidine value (AV) index were emploied to evaluate the antioxidant activity of EGCG and acetylated EGCG mixture^5^. The results of acetylation of EGCG by Zhu indicated that the antioxidant activity of acetylated EGCG mixture was lower than EGCG^9^. The lipophilicity of the tested compound was in the order tri-acetylated derivatives > di-acetylated derivatives > mono-acetylated derivatives^18^. Comparing with the previous studies, we found that the total antioxidant capacity, scavenging superoxide anion radical capacity, scavenging hydroxyl free radical capacity of SoEGCG were also significantly lower than EGCG. But the scavenging superoxide anion radical capacity of ToEGCG was significantly higher than EGCG. And the total antioxidant capacity of ToEGCG was significantly higher than EGCG. The scavenging superoxide anion radical of SoEGCG was higher than ToEGCG.

The possible reason is that the antioxidant activity is not only related to the number of substitution group, but also the space conformation. Though the poor solubility, easy conversion, low bioavailability of EGCG in utilization can be solved by acetylating, antioxidant activity of different EGCG acetylating components are influenced by different factors, e.g. stereo chemical structure, configuration. The influence mechanism still needs to be further discussed.

### Supplementary Information

Supplementary Information.

Supplementary Figure S1.

## Data Availability

The datasets used and/or analysed during the current study available from the corresponding author on reasonable request.
