# Supplementary material for: Separation and antioxidant activities of new acetylated EGCG compounds
Source: Sci Rep. 2023 Nov 28;13:20964. doi: 10.1038/s41598-023-48387-9 (PMC10684485; doi:10.1038/s41598-023-48387-9)
Supplement: Supplementary file 1 — Supplementary Information. [file 41598_2023_48387_MOESM1_ESM.doc]

The molecular weight of Fr.1 was 458 m/z. It is EGCG.


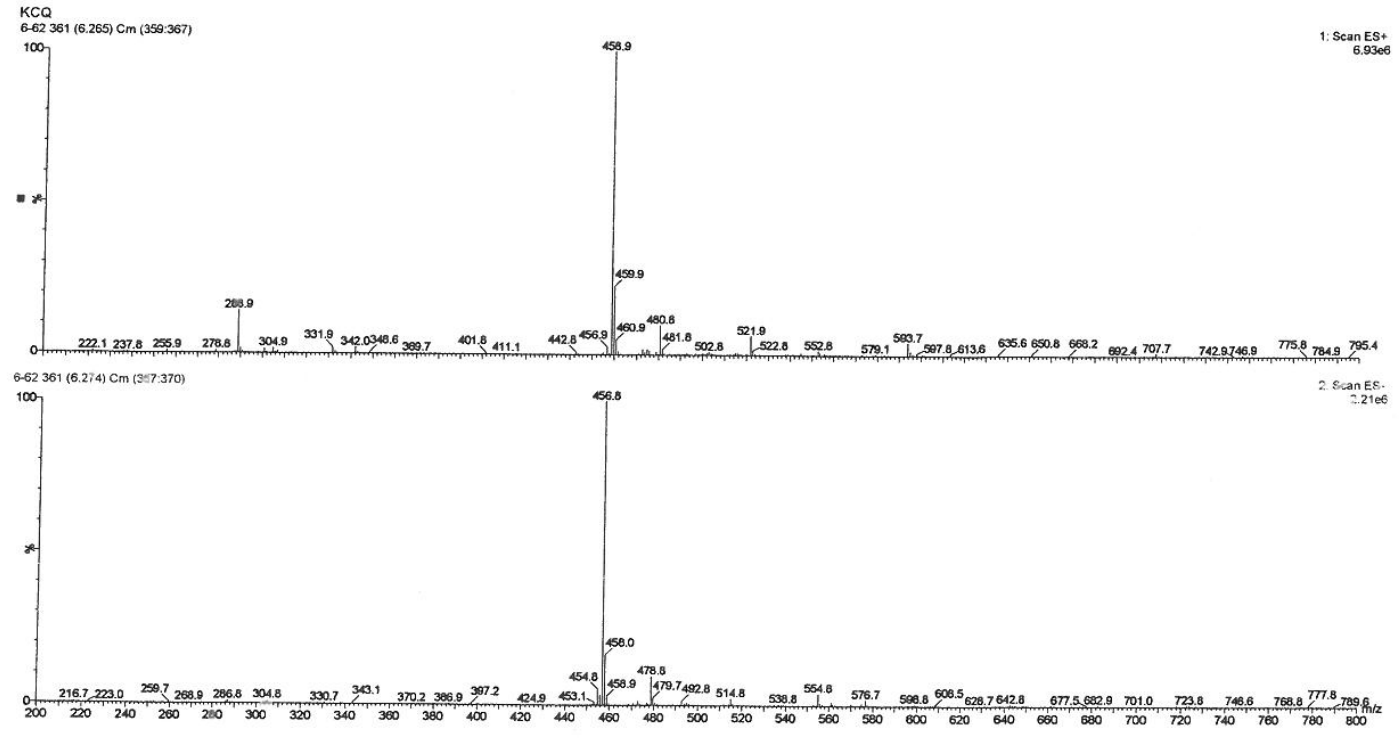


Mass spectrometry of EGCG (Fr.1)

The molecular weight of Fr.2 was 500 m/z.

Fr.2 is a single substituted O-acetylated epigallocatechin gallate (SoEGCG), which including 5-Acetyl- (-) –EGCG and 7-Acetyl- (-) –EGCG


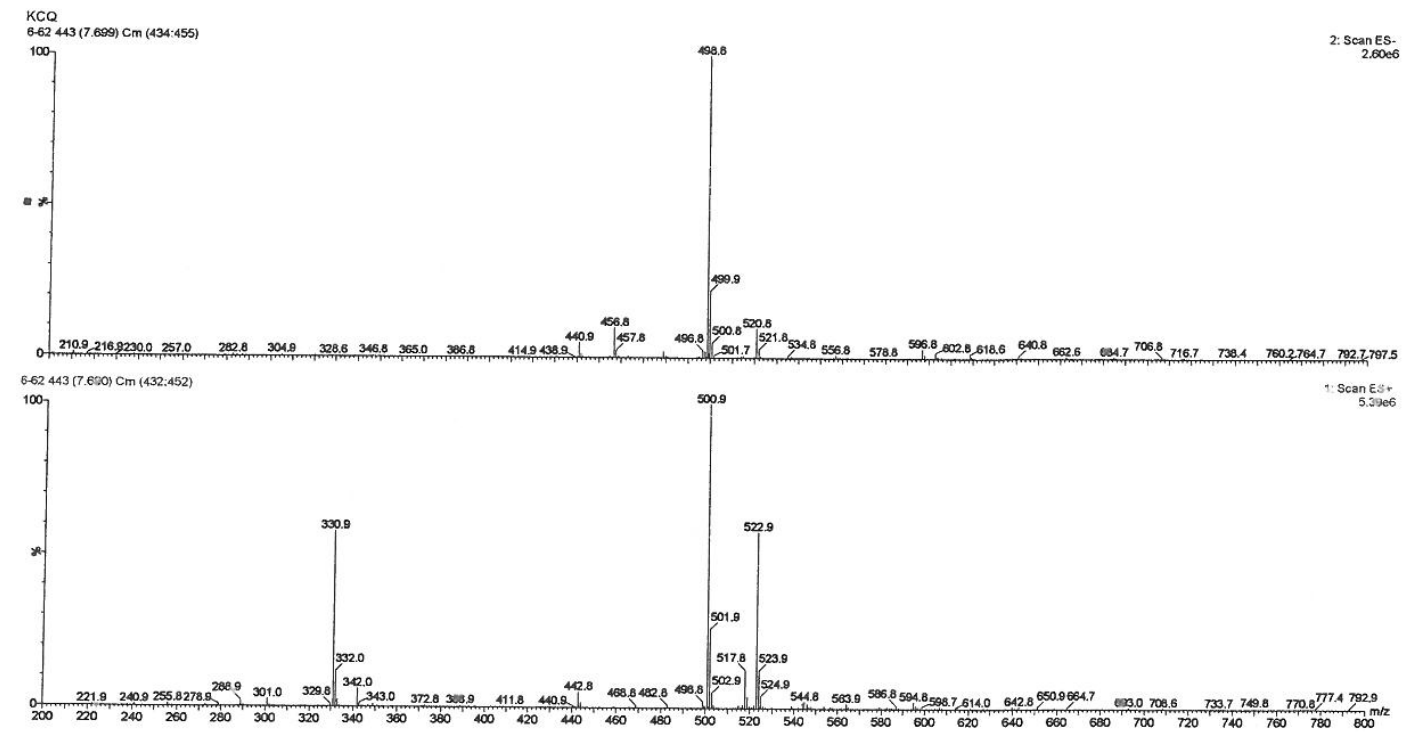


Mass spectrometry of SoEGCG (Fr.2)

**^13^C-NMR and ^1^H-NMR of 5-Acetyl- (-) –EGCG and 7-Acetyl- (-) –EGCG**


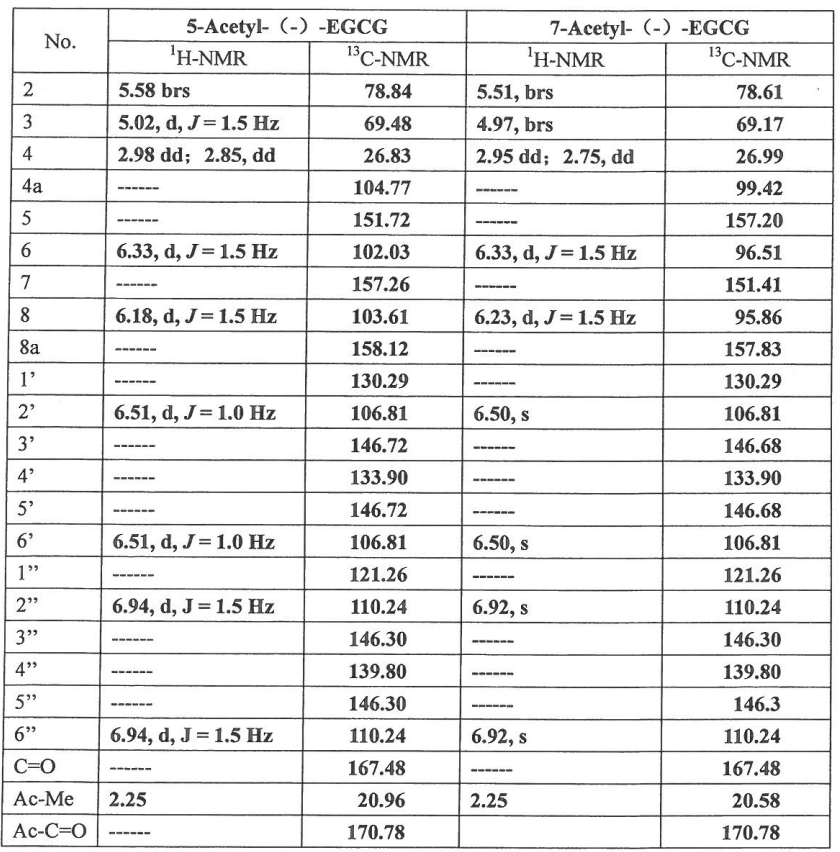


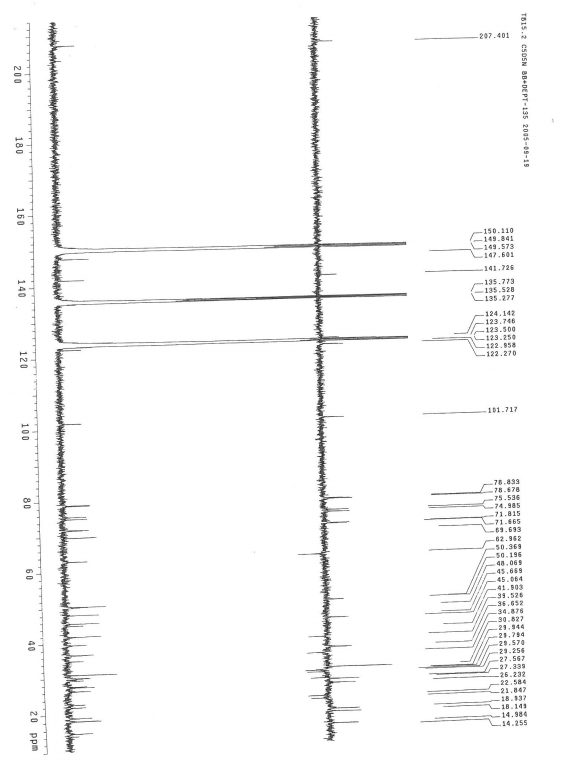


NMR of SoEGCG (Fr.2)

The molecular weight of Fr.3 was 484 m/z.

Fr.3 is a three substituted O-acetylated epigallocatechin gallate (ToEGCG).


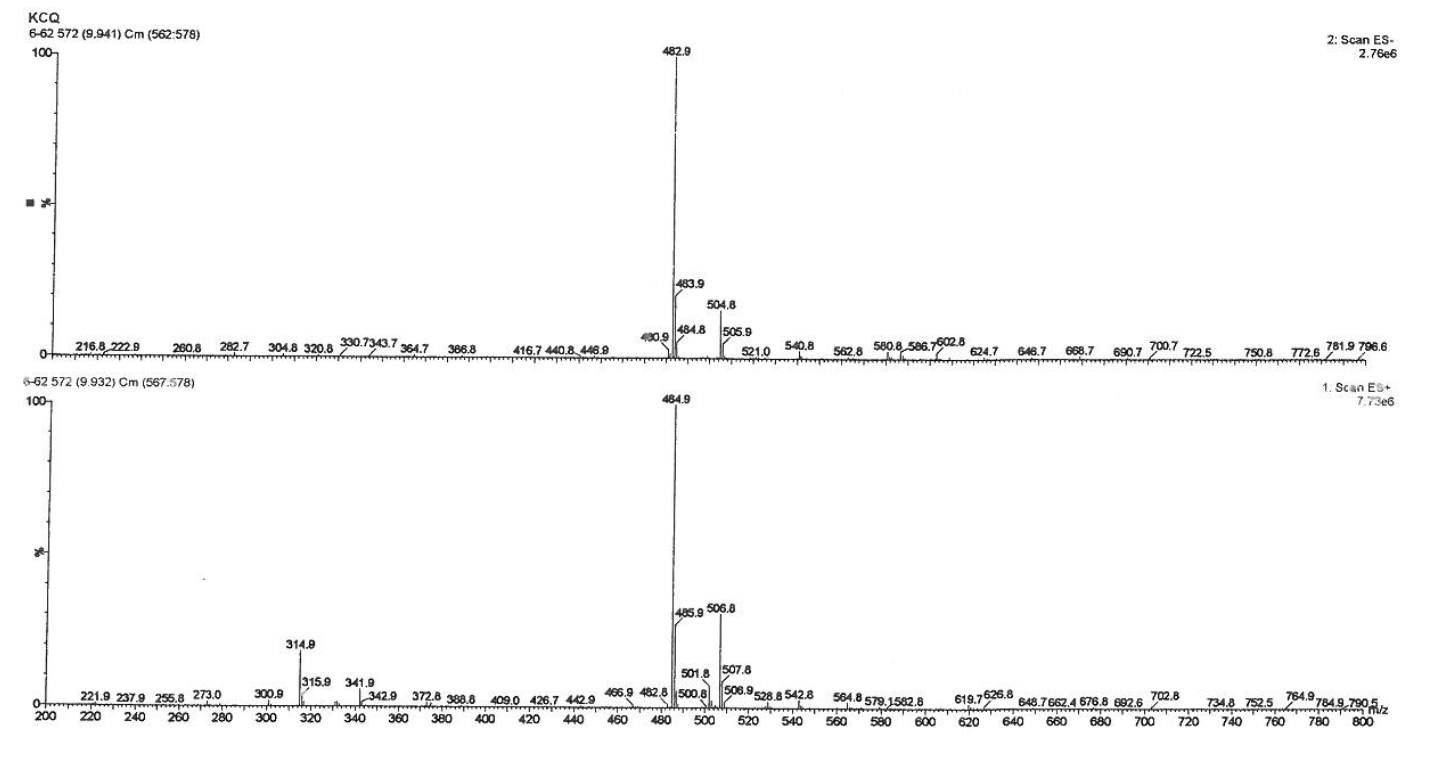


Mass spectrometry of TOEGCG (Fr.3)

The molecular weight of Fr.4 was 794 m/z.

Fr.4 is a eight substituted peracetylated epigallocatechin gallate (Ep-EGCG, E-AcE).


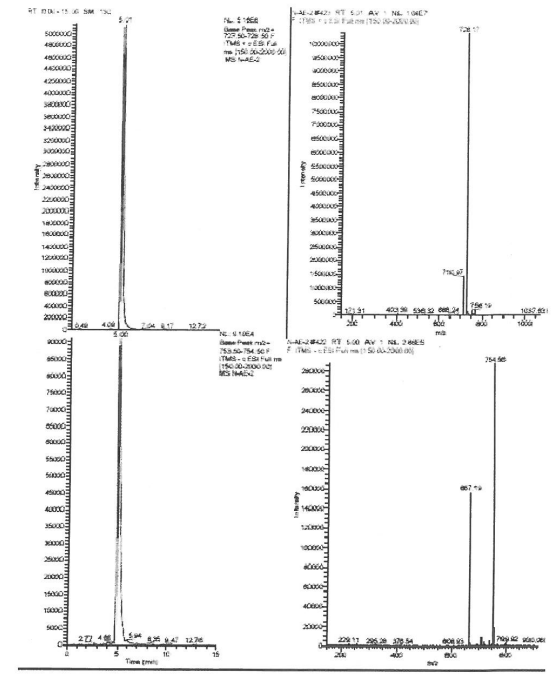


Mass spectrometry of ACEGCG (Fr.4)
