# Supplementary figures and images for: Separation and antioxidant activities of new acetylated EGCG compounds
Source: Sci Rep. 2023 Nov 28;13:20964. doi: 10.1038/s41598-023-48387-9 (PMC10684485; doi:10.1038/s41598-023-48387-9)

Supplementary material


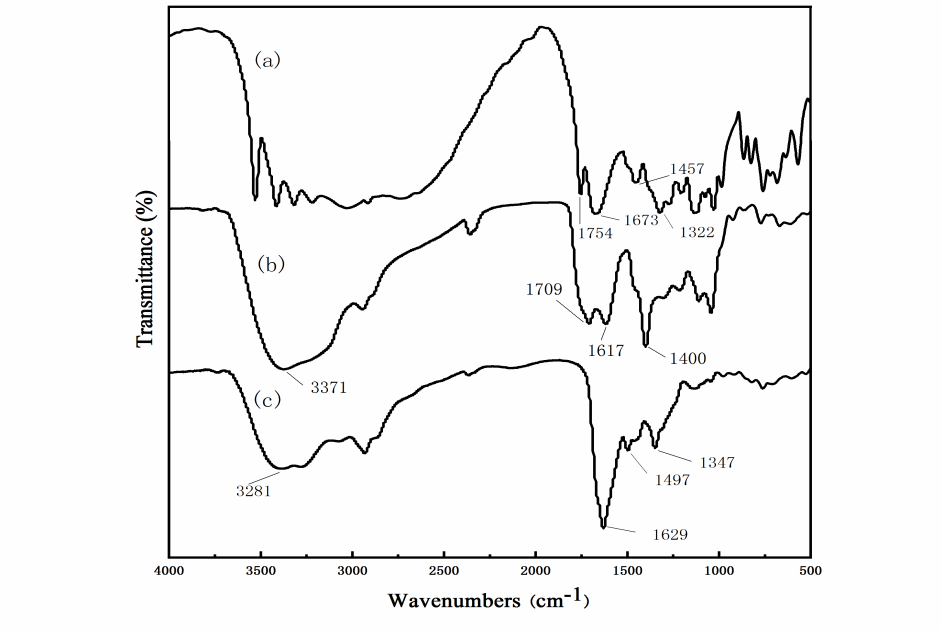


Fig s1. Infrared spectroscopy of (a)SoEGCG, (b)ToEGCG and (c)AcEGCG

Supplement: Supplementary file 2 — Supplementary Figure S1. [file 41598_2023_48387_MOESM2_ESM.doc]
